# Scavenging of reactive oxygen species by astaxanthin inhibits epithelial–mesenchymal transition in high glucose-stimulated mesothelial cells

**DOI:** 10.1371/journal.pone.0184332

**Published:** 2017-09-19

**Authors:** Kazuaki Hara, Chieko Hamada, Keiichi Wakabayashi, Reo Kanda, Kayo Kaneko, Satoshi Horikoshi, Yasuhiko Tomino, Yusuke Suzuki

**Affiliations:** 1 Department of Nephrology, Juntendo University Faculty of Medicine, Bunkyo, Tokyo, Japan; 2 Ikegami General Hospital, Tokyo, Japan; University of South Alabama Mitchell Cancer Institute, UNITED STATES

## Abstract

**Background:**

High glucose concentrations influence the functional and structural development of the peritoneal membrane. We previously reported that the oral administration of astaxanthin (AST) suppressed peritoneal fibrosis (PF) as well as inhibited oxidative stress, inflammation, and epithelial–mesenchymal transition (EMT) of peritoneal mesothelial cells (PMCs) in a chlorhexidine-induced PF rat model. This suggests that oxidative stress induction of EMT is a key event during peritoneal damage. The present study evaluated the therapeutic effect of AST in suppressing EMT, in response to glucose-induced oxidative stress.

**Methods:**

Temperature-sensitive mesothelial cells (TSMCs) were cultured in the presence or absence of AST and then treated with 140 mM glucose for 3 or 12 hours. Expression levels of TNF-α, TGF-β, and VEGF were determined at the mRNA and protein levels, and nuclear factor kappa B (NF-κB) activity was evaluated. We measured NO_2_^−^/NO_3_^−^ concentrations in cellular supernatants and determined 8-hydroxy-2′-deoxyguanosine (8-OHdG) levels in mitochondrial and nuclear DNA. The expressions of E-cadherin and alpha-smooth muscle actin (α-SMA) were evaluated by double immunofluorescence and protein levels.

**Results:**

High glucose concentrations induced overproduction of reactive oxidative species (ROS), increasing 8-OHdG mitochondrial DNA and cytokine levels. The NF-κB pathway was activated in response to high glucose concentrations, whereas *de novo* α-SMA expression was observed with decreased E-cadherin expression. AST treatment attenuated ROS production, inflammatory cytokine production, NF-κB activation, and EMT.

**Conclusion:**

The findings of the present study indicate that AST may have an anti-EMT effect due to anti-oxidative and anti-inflammatory activities by scavenging glucose-induced ROS from mitochondria in PMCs. AST may be an efficacious treatment for PF.

## Introduction

Peritoneal dialysis (PD) is an attractive treatment option for patients with end-stage kidney disease. Preservation of residual renal function and quality of life are the main advantages of PD. However, over the long term, PD patients develop functional and morphological alterations in the peritoneal membrane [1], limiting treatment benefits. The cumulative exposure of the peritoneum to unphysiologically high concentrations of glucose induces morphological changes in the peritoneum, including mesothelial cell loss, vasculopathy, and fibrotic thickening of the peritoneal interstitium [2–4]. The epithelial–mesenchymal transition (EMT) plays a crucial role in peritoneal fibrosis (PF) [5]. Previous reports indicate that glucose degradation products and advanced glycation end products induce functional and morphological peritoneal alterations [6], with alterations also observed in some patients during early phase PD therapy. Ciszewics *et al*. reported that high glucose concentrations accelerate aging in human peritoneal mesothelial cells (PMCs) [7]. Therefore, high glucose concentrations may directly injure mesothelial cells. Recently, neutral PD solutions have been shown to suppress the development of peritoneal morphological alterations [8]; however, high concentrations of glucose remain with the use of osmotic agents in PD solutions. It is important to evaluate the process of PF in response to high glucose concentrations to develop novel treatments. High glucose concentrations lead to the production of electrical potentials within the mitochondrial electron transfer system and production of O_2_^-^ at the intracellular membrane [9]. Nishikawa *et al*. posited that ROS production, in response to high concentrations of glucose, may contribute to several complications of diabetes mellitus [10]. We previously reported that the oral administration of astaxanthin (AST) diminished PF in chlorhexidine-induced PF rats [11]. AST is a prevalent carotenoid, with a polar structure at either end of the molecule, and a potent capacity for quenching ROS due to the presence of two oxygenated groups. Further, the structure of AST allows stable binding over the polar-nonpolar-polar span of the cell membrane [12]. ROS scavenging in the cellular membrane is the major anti-oxidative mechanism of AST. Therefore, we consider ROS to be a non-specific but crucial factor in peritoneal injury, particularly PF. We hypothesized that unphysiologically high concentrations of glucose in PD solution directly induce ROS in PMCs as an early reaction, with ROS then inducing EMT via several pathways. Therefore, the present study evaluated the therapeutic effect and underlying mechanisms of AST in suppressing EMT in response to glucose-induced oxidative stress.

## Materials and methods

### Preparation of temperature-sensitive mesothelial cells

We used stocked temperature-sensitive mesothelial cells (TSMCs) established from temperature-sensitive Simian virus 40 (SV40) large T-antigen gene transgenic rats ((pSVtsA58ori[−]-2) [13,14]), which enable subculture passage and are frozen using liquid nitrogen [14,15]. TSMCs were progressively grown at 33°C and suspended and differentiated at 38°C, the temperature at which the cells continuously maintained the native morphological and functional characteristics of primary mesothelial cells. TSMCs were cultured on the surface of 100-mm culture dishes with a M199 medium (Invitrogen Co., Tokyo, Japan) containing 10% fetal bovine serum (FBS), 10 U/mL of penicillin, and 100 μg/mL of streptomycin at 33°C in a 5% CO_2_ incubator, as described previously [15]. The medium was replaced every 3 days. After reaching a density of 1 × 10^6^, the cells were further cultured at 38°C for 7 days in M199 medium to synchronize cellular quiescence. We have observed the ethical guidelines of our institution (the institutional animal care and use committee (IACUC) at Juntendo University Faculty of Medicine, Tokyo, Japan, Approval Number: 260158).

### Preparation of astaxanthin

The AST was provided by AstaReal Co., Ltd. (Tokyo, Japan). We dissolved 5 mmol (2.98 mg) of AST in 1 mL of dimethyl sulfoxide to prepare a 5 mM solution. The AST stock was stored at −80°C and then heated at 70°C for 5 min until activation.

### Experimental design

**Fig 1:**
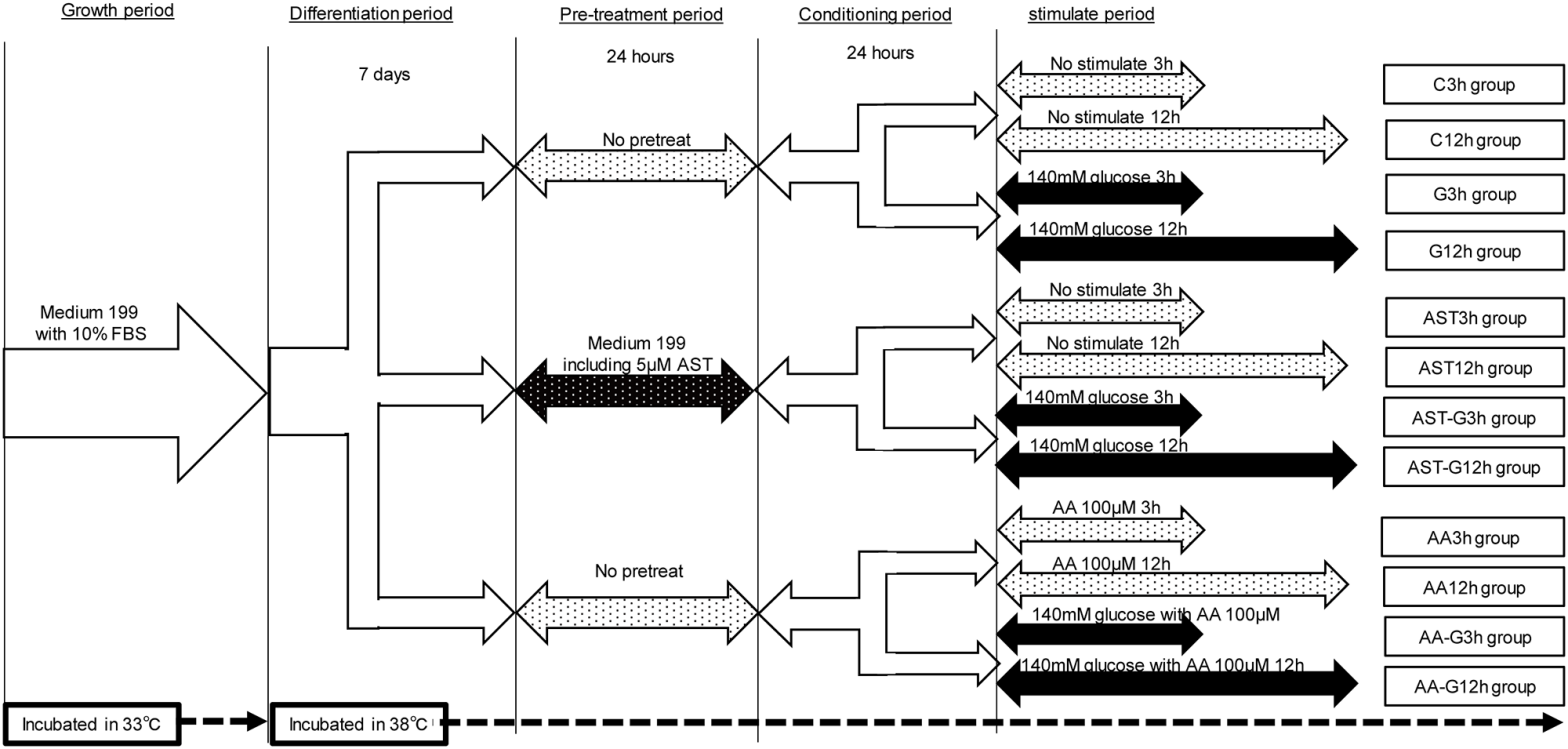
Study groups and experimental design. Unstimulated control groups (Control group): Incubated in M199 medium alone for 3 h (C3h group) or 12 h (C12h group). G groups: Incubated in M199 medium with 140 mM glucose for 3 h (G3h group) or 12 h (G12h group). Ascorbic acid (AA; AA group.): Incubated in M199 medium with 140 mM glucose and 100 μM AA for 3 h (AA-G3h group) or 12 h (AA-G12h group). AST pre-treated groups (AST group): Incubated in M199 medium alone for 3 h (AST3h group) or 12 h (AST12h group) after incubated in M199 medium with 5μM AST for 24h. AST-G group: Incubated in M199 medium with 140 mM glucose for 3 h (AST-glucose [AST-G] 3h group) or for 12 h (AST-G12h group) after incubated in M199 medium with 5μM AST for 24h.

Synchronized cells were cultured and divided into 2 groups: M199 medium with 10% FBS alone (Medium alone group) or medium pre-treated with AST for 24 h (AST pre-treated group). Pre-treated TSMCs were incubated for 24 h (conditioning period). Then, we divided TSMCs as follows (Fig 1). 1) Medium alone groups: In the unstimulated control group (Control group), cells were incubated in M199 medium alone. In all other groups, cells were incubated with 140 mM glucose for 3 h (G3h group) or 12 h (G12h group). We also stimulated cells in 140 mM glucose with ascorbic acid (AA; AA group; Wako pure chemical industries, Ltd, Osaka, Japan), which has a potent anti-oxidative capacity as previously described [16, 17]. We determined the concentration according to previously published reports [17–19] for 3 h (AA- glucose [AA-G] 3h group) or 12 h (AA-G12h group) as contrast to the anti-oxidant effects of AST. 2) AST pre-treated groups: In the unstimulated control group (AST group), cells were incubated in M199 medium containing AST. Once again, we determined concentrations according to previously published reports [20]. In all other groups, cells were incubated in the presence of 140 mM glucose for 3 h (AST-glucose [AST-G] 3h group) or for 12 h (AST-G12h group) (Fig 1). After the stimulation period, cells were harvested by trypsin—EDTA digestion at the indicated time points and counted using a hemocytometer.

### Determination of the NO_2_^−^/NO_3_^−^ levels in supernatants, intracellular ROS levels, and 8-hydroxy-2′-deoxyguanosine levels in mitochondrial and nuclear DNA

To evaluate the anti-oxidative effect of AST, NO_2_^−^/NO_3_^−^ (nitrite/nitrate) levels in cell supernatants and intracellular ROS levels were measured, and we determined the 8-hydroxy-2′-deoxyguanosine (8-OHdG) levels in mitochondrial and nuclear DNA. The levels of NO_2_^−^/NO_3_^−^ in cell supernatants were measured using the NO_2_^−^/NO_3_^−^ Assay Kit-C II (NK05, Dojindo Molecular Technologies, Inc., Kumamoto, Japan). Cell supernatants were deproteinized using an Amicon Ultra-4 centrifugal filter units with Ultracel-10 membranes (Millipore, Billerica, MA, USA), and NO_2_^−^/NO_3_^−^ levels were measured according to the manufacturer’s instructions. Before the comparison study, we examined the anti-oxidative effects of AST and AA in a dose-dependent study. In AST and AA groups, up to 5 μM AST and 100 μM AA significantly suppressed the NO_2_^−^/NO_3_^−^ levels compared with glucose alone. We then established AST and AA concentrations as 5 μM and 100 μM, respectively (S1 Fig). Intracellular ROS levels were measured using the OxiSelect^™^ Intracellular ROS Assay Kit (Green Fluorescence; Cell Biolabs, Inc. San Diego, CA, USA) according to the manufacturer’s instructions. We cultured TSMCs in a 96-well cell culture plate until well was confluent. We added 100 μL of 1 mM 2′, 7′-dichlorodihydrofluorescin diacetate (DCFH-DA) solution in culture medium to TSMCs and incubated the mixture at 37°C for 60 min. After DCFH-DA treatment, TSMCs were analyzed on an inverted fluorescence plate reader, using excitation and emission wavelengths of 480 nm and 530 nm, respectively. A standard curve was made using 2′,7′-dichlorodihydrofluorescein (DCF) standard solution (the manufacture’s solution). We directly observed the levels of fluorescence using the fluorescent microscope BZ-X700 (Keyence, Osaka, Japan). We determined 8-OHdG levels in mitochondrial and nuclear DNA using the Epiquick 8-OHdG DNA Damage Quantification Direct Kits (Epigentek, New York, NY, USA) according to the manufacturer’s instructions. Optical density (OD) was measured at 540 nm using a plate reader. Mitochondrial DNA was isolated using mitochondrial DNA isolation kits (BioVision, Milpitas, CA, USA) and Dounce homogenizer (Wheaton Industries Inc., Millville, NJ, USA) according to the manufacturer’s instructions. Nuclear DNA was isolated using NucleoSpin^®^ Tissue (Macherey-Nagel, Inc., Germany) according to the manufacturer’s instructions.

### Measurement of cytokine and growth factor mRNA levels by real-time reverse transcription-polymerase chain reaction

RNA extraction and real-time polymerase chain reaction (PCR) analysis were performed as previously described [15]. Total RNA was extracted from peritoneal specimens using TRIzol^®^ reagent (Invitrogen AG, Basel, Switzerland) and RNeasy Mini Kits (Qiagen K.K., Tokyo, Japan). We determined RNA quantity using a NanoDrop ND-1000 spectrophotometer (NanoDrop Products, Wilmington, DE, USA). A 16 μL reaction mixture containing 1 μg of RNA, 4 μL of 2.5 mmol/L dNTP mixture (Takara Bio, Inc., Shiga, Japan) and 2 μL of Random Decamers RETRO script (Ambion, Inc., Austin, TX, USA) in RNase-free water was inactivated by heating at 70°C for 3 min. The product was added to 2 μL of 10× PCR buffer (Takara Bio, Inc., Shiga, Japan), 1 μL of Protector RNase Inhibitor (Roche Diagnostics Corp., Mannheim, Germany), and 0.5 μL of M-MLV Reverse Transcriptase (Invitrogen Corp., Carlsbad, CA, USA), then incubated at 42°C for 60 min. The complementary DNA (cDNA) product was used for real-time PCR. A 2 μL aliquot of diluted cDNA, 1.6 μL forward primers, 1.6 μL reverse primers, 10 μL SYBR Green PCR Master Mix (Applied Biosystems, Carlsbad, CA, USA), and 4.8 μL of cDNA-free double-distilled water were combined to form a final reaction mixture of 20 μL, according to the manufacturer’s instructions. The mixture was denatured and amplified using a 7500 Real-Time PCR system (Applied Biosystems) under the following conditions: (i) 20s at 95°C for 1 cycle, (ii) 3s at 95°C and 30 s at 60°C for 40 cycles, (iii) 15s at 95°C, 60s at 60°C, 15s at 95°C, and 15s at 60°C for 1 cycle. cDNA-free double-distilled water was included in each reaction as a negative control. For the quantification of PCR products, samples were standardized for glyceraldehydes-3- phosphate dehydrogenase. PCR primers are shown in S1 Table.

### Measurement of TGF-β, VEGF, and TNF-α in supernatants

Tumor necrosis factor-α (TNF-α), transforming growth factor-β (TGF-β), and vascular endothelial growth factor (VEGF) levels were determined in cell supernatants by enzyme-linked immunosorbent assay (ELISA) using TNF-α, TGF-β, and VEGF Quantikine Rat immunoassay kits (R&D Systems, Minneapolis, MN, USA) according to the manufacturer’s instructions. OD was measured at 450 nm using a plate reader.

### Evaluation of epithelial–mesenchymal transition

Double immunofluorescence for E-cadherin and alpha-smooth muscle actin (α-SMA) was performed as described previously [21]. TSMCs (1.0 × 10^5^), incubated on culture cover glasses in 6-well plates, were washed once with phosphate-buffered saline (PBS) and placed in methanol for 5 min for fixation, followed by 3 washes with PBS. The cells were then incubated with a Carbo-Free Blocking Solution (Vector Laboratories, Inc., Burlingame, CA, USA) and further incubated with a mouse anti-rat E-cadherin antibody diluted to 1:100 (Abcam, Cambridge, MA, USA), reactive to epithelial cells, and a rabbit anti-rat α-SMA antibody diluted to 1:100 (Abcam), reactive with fibroblasts, for 60 min. Cells were washed 5 times with PBS and mounted in diluted Alexa 488 green or Alexa 555 red as a secondary antibody at room temperature for 60 min. Following the 1-h incubation with the secondary antibody, samples were washed 5 times with PBS for nuclear staining. The samples were stored at 4°C overnight, and we observed expression levels of E-cadherin and α-SMA under a confocal microscope (FV1000; Olympus, Tokyo, Japan). Negative control staining was performed by omitting the primary antibodies. In all fluorescent images, cell nuclei were labeled with a 4′,6-diamidino-2-phenylindole (DAPI) stain. The mRNA levels of E-cadherin and α-SMA were measured with real-time RT-PCR, and proteins were semi-quantitatively measured with Western blotting. Western blot method was as follows: 1.0 × 10^6^ TSMCs were grown on a 100-mm dish for confluence; cells were pelleted by centrifugation and washed twice with ice-cold PBS. TSMCs were re-suspended in 0.1 mL of RIPA buffer (Santa Cruz Biotechnology Inc, Dallas, TX, USA) and incubated on ice for 30 min to induce cell lysis. Samples were resolved on SDS polyacrylamide gels and then transferred to membranes and blocked with 5% non-fat milk solution. These were subsequently incubated with the appropriate primary antibodies. Antibodies against E-cadherin (abcam) and α-SMA (abcam) were applied at a 1:300 dilution, and HRP-conjugated secondary antibodies (Promega, Madison, WI, USA) were applied at a 1:10000 dilution. We scanned all images using a LAS-3000 (FUJIFILM, Tokyo, Japan).

### Evaluation of nuclear factor kappa B activity in the nucleus

The nuclear levels of the nuclear factor kappa B (NF-κB) p65 subunit were measured using NF-κB/p65 ActivELISA kits (Novus Biologicals, Littleton, CO, USA), according to the manufacturer’s instructions. Nuclear fractions from cultured TSMCs were extracted according to the lysate preparation manual. We used anti-p65 antibody-coated plates to capture free p65 from the nuclear lysates, and the amount of bound p65 was detected by adding a secondary anti-p65 antibody, followed by an alkaline phosphatase-conjugated detection antibody. Para-nitrophenyl phosphate was used as a substrate for colorimetric detection, performed at OD 405 nm using a plate reader. Immunofluorescence of the p65 subunit was detected as follows. TSMCs (1.0 × 10^5^) incubated on culture cover glasses in 6-well plate were washed once with PBS and placed for 5 min in methanol for fixation, followed by three washes with PBS. The cells were then incubated with a Carbo-Free Blocking Solution (Vector Laboratories) and further incubated with a mouse anti-rat NF-κB p65 antibody diluted at 1:100 (Abcam) for 60 min. The cells were washed 5 times with PBS and mounted in diluted Alexa 488 green as a secondary antibody at room temperature for 60 min. Samples were stored at 4°C overnight, and the expression levels of the p65 subunit were observed under a confocal microscope FV1000. We performed negative control staining of the samples by omitting the primary antibody. Western blotting was performed as previously described. NF-κB antibody (abcam) were used at a 1:300 dilution.

### Statistical analysis

Data were expressed as the mean ± standard deviation (SD). Differences between groups were examined for statistical significance using Student’s *t*-test. Statistical analyses were performed sing GraphPad Prism version 6.0 software (MDF, Tokyo, Japan). P-values of <0.05 were considered statistically significant.

## Results

### Effect of high glucose stimulation, AST, and AA to TSMCs as for ROS

**Fig 2:**
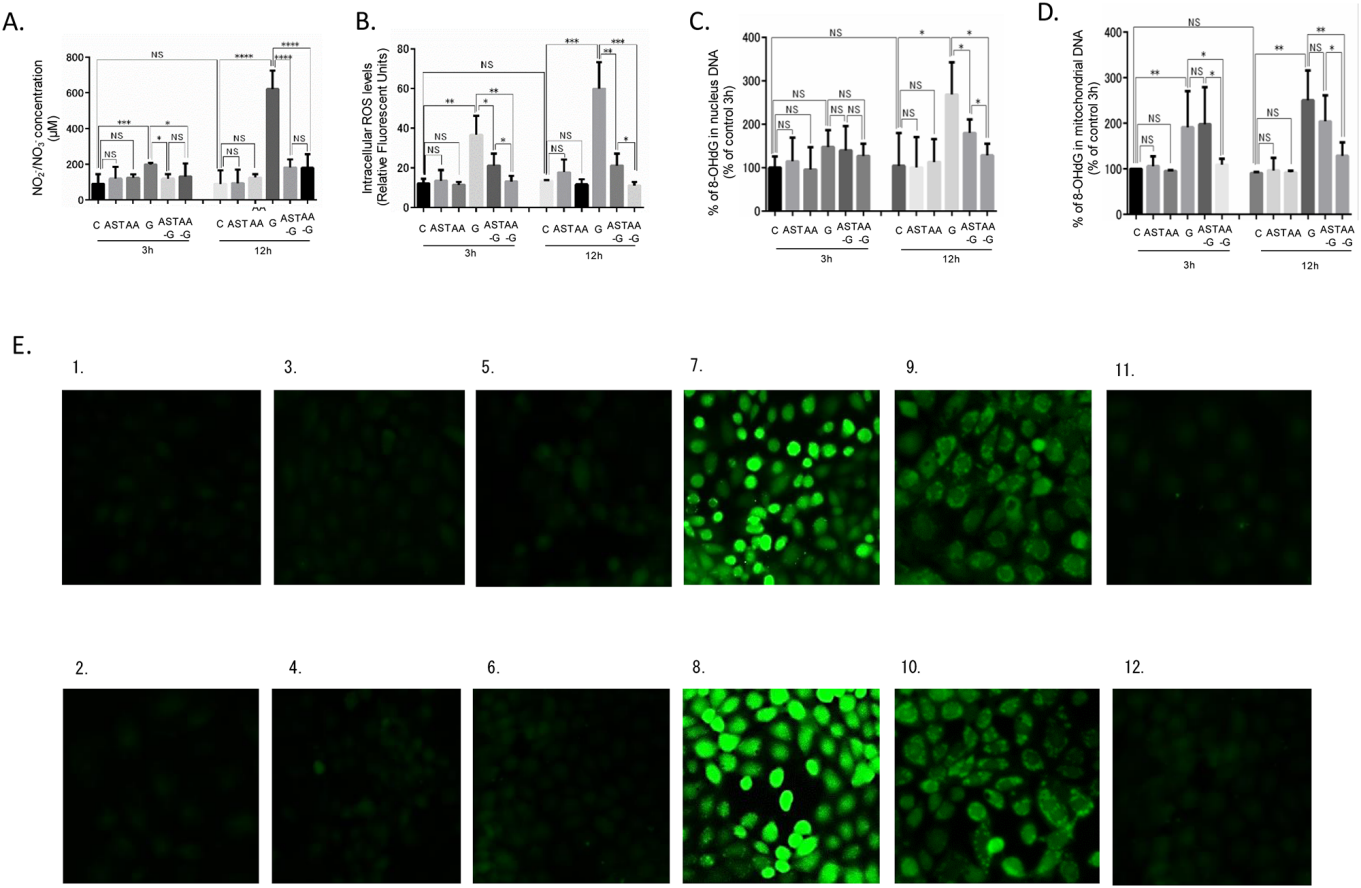
Effect of high glucose stimulation, AST, and AA to TSMCs as for ROS. (A) NO_2_^−^/NO_3_^−^ concentration in medium supernatant of each group. (B) Intracellular ROS levels of each group. (C) 8-OHdG ratio in mitochondrial DNA of each group. (D) 8-OHdG ratio in nuclear DNA of each group. AST concentration: 5 μM, AA concentration: 100 μM. NS: no significant change. *: p < 0.05. **: p < 0.01. ***: p < 0.0005. ****: p < 0.0001. Error bars represent SD. (E) ROS fluorescence of each group (1) C3h group, (2) C12h group, (3) AST3h group, (4) AST12h group, (5) AA3h group, (6) AA12h group, (7) G3h group, (8) G12h group, (9) AST-G3h group, (10) AST-G12h group, (11) AA-G3h group, and (12) AA-G12h group. Fluorescence solution was 2′, 7′-dichlorodihydrofluorescin diacetate (DCFH-DA).

Total NO_2_^−^/NO_3_^−^ and intracellular ROS levels in the G3h and G12h groups were significantly higher than those in the control group, with total NO_2_^−^/NO_3_^−^ increasing with incubation time (Fig 2A and 2B). 8-OHdG levels in mitochondrial DNA in each glucose-stimulated group were significantly higher than in the unstimulated group (Fig 2C). 8-OHdG levels in nuclear DNA in the G12h group were significantly higher than in the control group (Fig 2D). AST pre-treatment suppressed NO_2_^−^/NO_3_^−^ elevations that were induced by glucose. NO_2_^−^/NO_3_^−^ levels in AST groups were comparable with those in the control group (Fig 2A). Intracellular ROS levels in AST-G groups were significantly lower than the G groups at 3 and 12 h (Fig 2B), but intracellular ROS in AST-G groups was significantly higher than that of AA-G groups (Fig 2B). In addition, AST did not suppress the production of 8-OHdG in mitochondrial and nuclear DNA (Fig 2C and 2D). AA suppressed total NO_2_^−^/NO_3_^−^ as well as AST and intracellular ROS levels compared with AST (Fig 2B). 8-OHdG levels in both mitochondrial and nuclear DNA significantly decreased (Fig 2C and 2D). In fluorescent analysis of ROS, fluorescence was observed in whole cells in the G groups. In the AST groups, ring-shaped fluorescence was observed along the cell membrane (Fig 2E). There were no significant changes in ROS levels in the C, AST, and AA alone groups (Fig 2E).

### Effect of high glucose stimulation, AST, and AA to TSMCs as for cytokine

**Fig 3:**
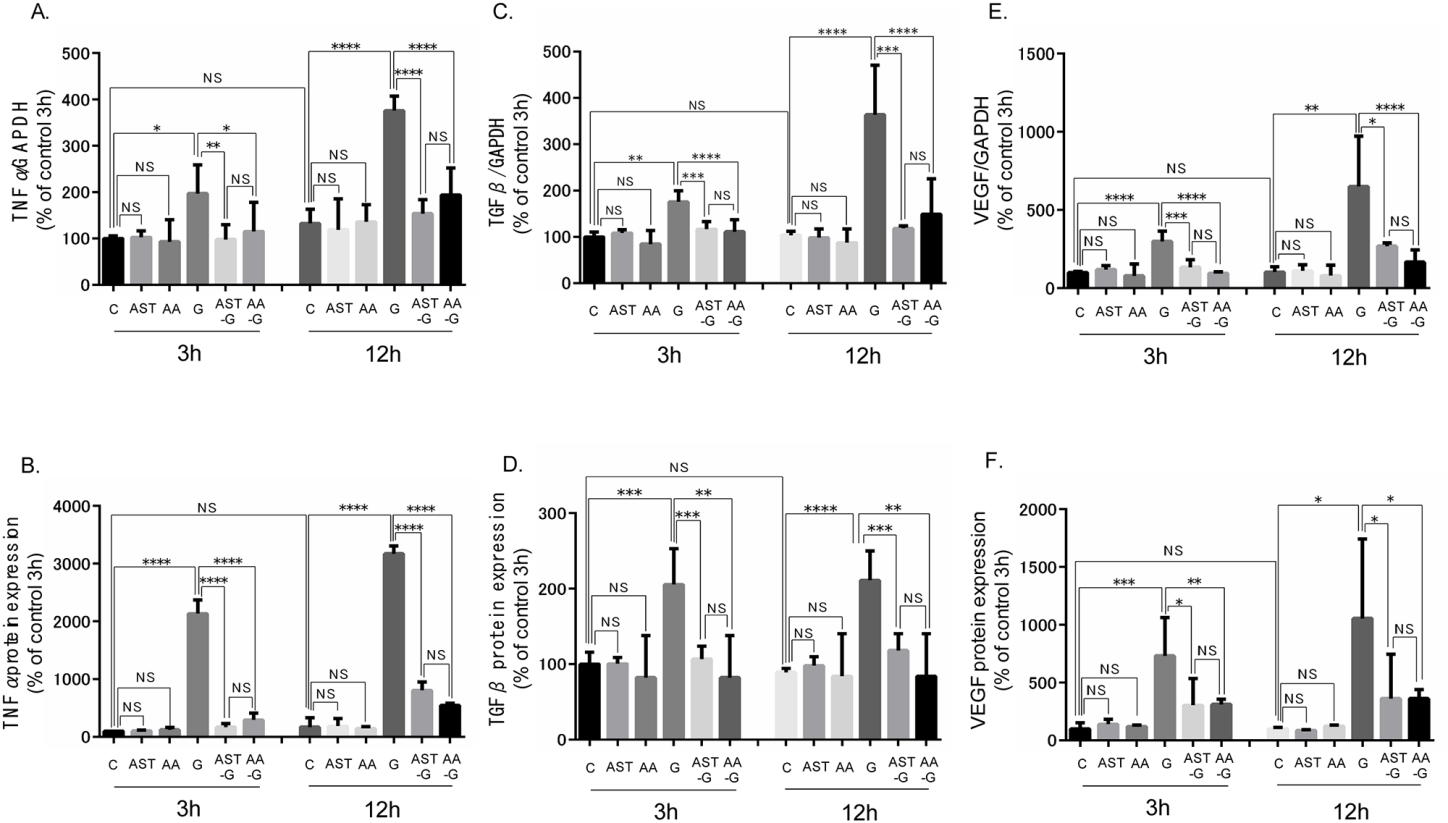
Effect of high glucose stimulation, AST, and AA to TSMCs as for cytokines. (A and B) Expression of TNF-α mRNA and protein levels in each group. (C and D) Expression of TGF-β mRNA and protein levels in each group. (E and F) Expression of VEGF mRNA and protein levels in each group. *: p < 0.05. **: p < 0.01. ***: p < 0.0005. ****: p < 0.0001. Error bars represent SD.

High concentrations of glucose significantly stimulated mRNA and protein expression of TNF-α, TGF-β, and VEGF (Fig 3A–3F). AST pre-treatment and AA treatment attenuated increases in cytokine mRNA and protein expression levels in response to glucose (Fig 3A–3F), whereas cytokine mRNA and protein levels both in the AST and AA alone groups were comparable with those in the control group (Fig 3D–3F).

### Evaluation of EMT

**Fig 4:**
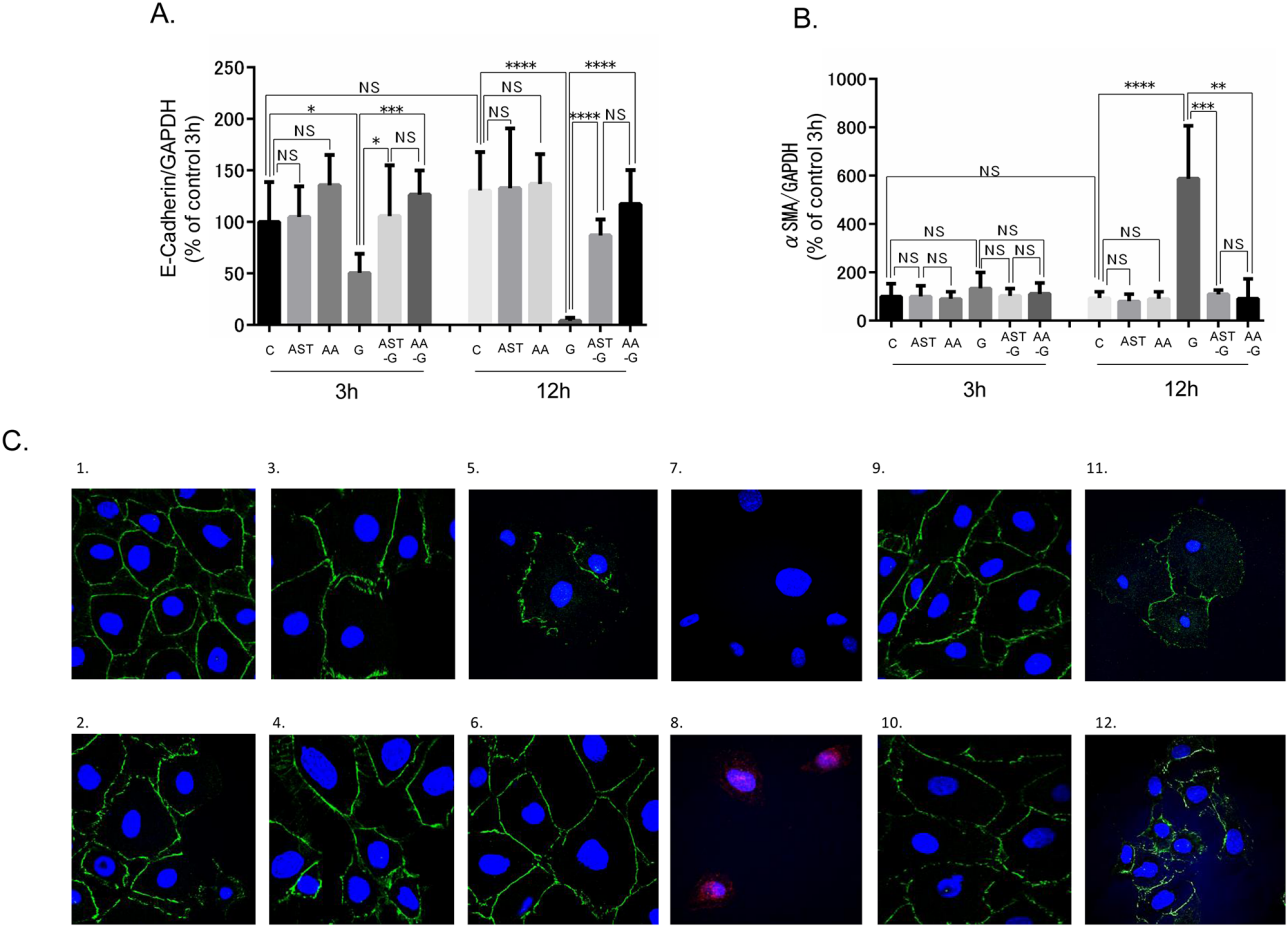
Effect of high glucose stimulation, AST and AA to TSMCs as for EMT. (A) E-cadherin mRNA and protein expression levels in each groups. (B) α-SMA mRNA and protein expression levels in each group. :p < 0.05. **: p < 0.01. ***: p < 0.0005. ****: p < 0.0001. Error bars represent SD. (C) Effect of high glucose stimulation, AST, and AA to TSMCs as for EMT in double immunofluorescence. (1) C3h group, (2) C12h group, (3) AST3h group, (4) AST12h group, (5) AA3h group, (6) AA12h group, (7) G3h group, (8) G12h group, (9) AST-G3h group, (10) AST-G12h group, (11) AA-G3h group, and (12) AA-G12h group. Alexa 488 green (E-cadherin) and Alexa 555 red (α-SMA) were used as secondary antibodies. DAPI was used for nuclear staining.

High concentrations of glucose significantly decreased E-cadherin mRNA and protein levels and increased α-SMA. The effects of glucose on E-cadherin and α-SMA expression were enhanced with treatment time (Fig 4A and 4B and S2 Fig). Immunofluorescence demonstrated that E-cadherin expression was maintained in the control group and diminished in the G3h and G12h groups (Fig 4A). Expression of mRNA levels of α-SMA was observed in the G12h group only (Fig 4B). AST pre-treatment and AA treatment completely inhibited the effects of glucose on E-cadherin and α-SMA mRNA and protein levels (Fig 4A and 4B). Immunofluorescence demonstrated that E-cadherin expression in AST and AA alone groups were similar to that in the C group; E-cadherin expression in G group diminished, and it was maintained in the AST-G and AA-G groups (Fig 4C) and α-SMA expressed in G12h group only (Fig 4C).

### Evaluation of the NF-κB signaling pathway

**Fig 5:**
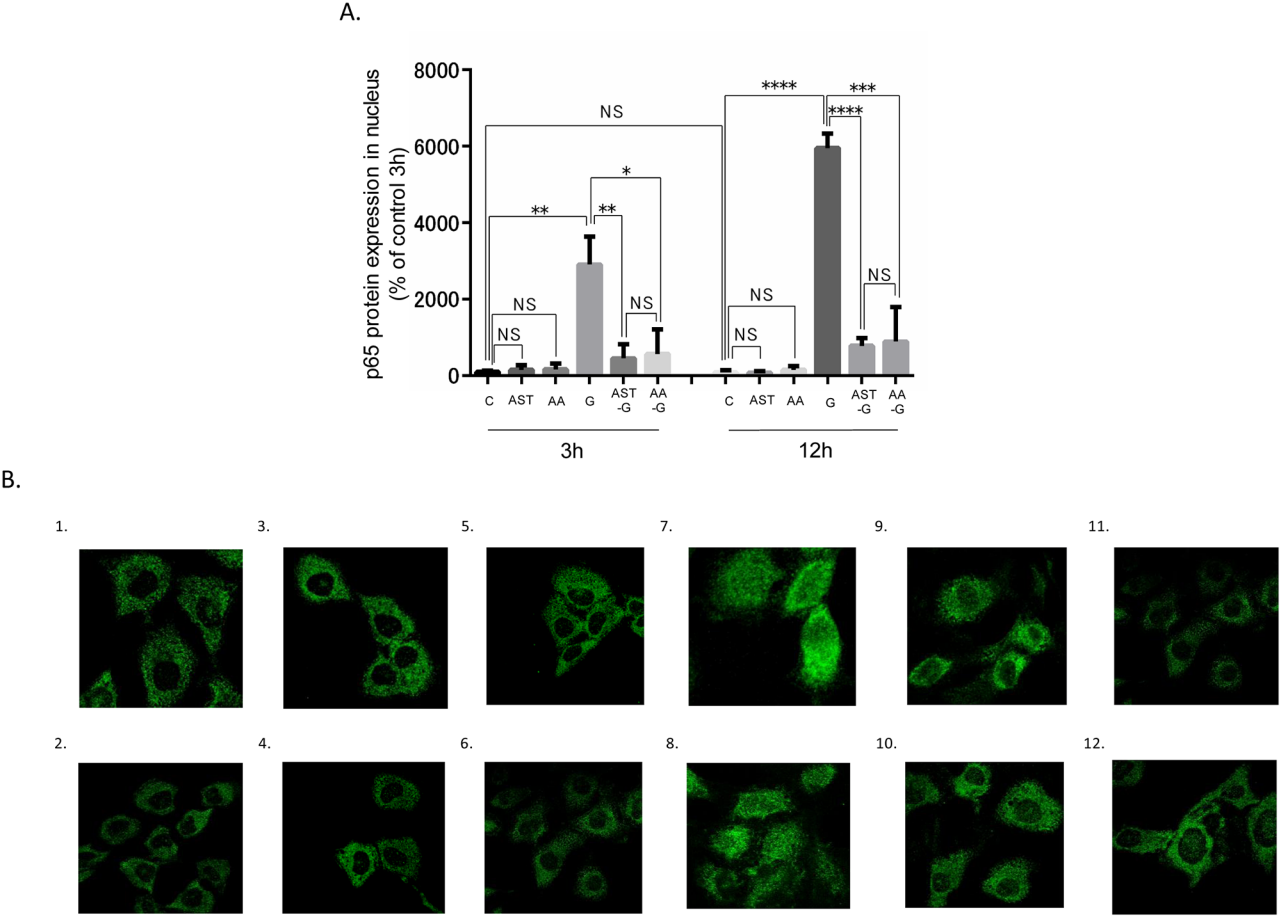
Effect of high glucose stimulation to TSMCs as for NF-κB pathway. (A) NF-κB p65 protein subunit expression in TSMCs in each group. *: p < 0.05. **: p < 0.01. ***: p < 0.0005. ****: p < 0.0001. Error bars represent SD. (B) NF-κB p65 subunit immunofluorescence. (1) C3h group, (2) C12h group, (3) AST3h group, (4) AST12h group, (5) AA3h group, (6) AA12h group, (7) G3h group, (8) G12h group, (9) AST-G3h group, (10) AST-G12h group, (11) AA-G3h group, and (12) AA-G12h group. Alexa 488 green were used as secondary antibodies.

Nuclear protein levels of nuclear p65, a subunit in the NF-κB signaling pathway, in the G groups significantly increased compared with those in the C group (Fig 5A and S2 Fig). Furthermore, although the NF-κB p65 subunit was observed in the cytoplasm in the C, AST, and AA alone groups, exclusively nuclear expression by immunofluorescence was observed in the G groups (Fig 5B). AST pre-treatment and AA treatment suppressed increased nuclear p65 protein expression (Fig 5A). Immunofluorescence demonstrated that the NF-κB p65 subunit was exclusively expressed in the cytoplasm in the AST-G and AA-G groups (Fig 5B).

## Discussion

The findings of the present study demonstrate that mitochondrial ROS produced in response to high glucose concentrations, similar to those of conventional PD solution, play a crucial role in EMT. In addition, to the best of our knowledge, this is the first study to demonstrate that AST inhibits EMT by suppressing ROS production in PMCs.

Our findings suggest ROS-induced EMT is mediated by several pathways, including those involving TGF-β and TNF-α. Immunohistochemical studies revealed a decrease in E-cadherin expression at the early stage of EMT, with α-SMA expression then detected. These results indicate that the EMT phenomenon develops in a step-by-step manner.

Mesothelial cells undergo EMT in response to continuous PD, and this process plays an active role in the structural and functional alterations observed in the peritoneal membrane [22]. PF is caused by EMT, and TGF-β production leads to EMT in the mesothelial cells of TGF-β transgenic PF mice [5]. TNF-α may also promote EMT in human renal cell carcinoma cell lines [23]. ROS and TNF-α produced by mesothelial cells under high glucose conditions promote IκB kinase (IKK) phosphorylation of IκBα protein, which results in ubiquitination, dissociation of IκBα from NF-κB, and eventual degradation of IκBα by the proteosome. Activated NF-κB is then translocated into the nucleus where it binds to specific sequences of DNA. The DNA/NF-κB complex then recruits other proteins, such as TGF-β and TNF-α [24]. The results of the present study also indicate that NF-κB activity is increased by glucose stimulation. Previous reports have also indicated that NF-κB activity maintains mesenchymal cellular state after EMT [25]. Non-physiological concentrations of glucose damage mitochondria due to ROS production leading to the formation of 8-OHdG in mitochondrial DNA. In the present study, 8-OHdG was significantly elevated in the mitochondria of the G3h and G12h groups, while 8-OHdG in the nucleus was only elevated in the G12h group. A previous study of a diabetic rat model demonstrated a high glucose concentration stimulated ROS production in the mitochondria but not in the nucleus [26], which suggests ROS produced in the mitochondria was the main cause of oxidative stress in mesothelial cells and oxidation of nuclear DNA was only a secondary effect. Fukasawa *et al*. [27] reported ROS-induced EMT through TGF-β1 secretion in normal human epidermal keratinocytes. Previous studies have reported high glucose-induced ROS directly lead to the production of cytokines such as TGF-β and TNF-α [27, 28]. The results of this study indicate the levels of ROS, cytokines, and NF- κB increase over time. Although E-cadherin was detected in G3h cells, α-SMA was observed only in G12h cells. According to these results, ROS produces cytokine, and cytokine leads to the stimulation of EMT in TSMCs. The present study suggests ROS produced in mitochondria under high glucose conditions may play a major role in the early phase of peritoneal injury.

AST is a prevalent carotenoid with a polar structure at either end of the molecule and a potent capacity for quenching ROS due to the presence of two oxygenated groups in each ring [29]. Kim *et al*. reported that AST has protective action against high-glucose-induced oxidative stress, inflammation, and apoptosis in proximal tubular epithelial cells [30]. The biological activity of AST was greater than other anti-oxidants due to its transmembranous nature, with its anti-oxidant activity shown to be 10 times greater than zeaxanthin, lutein, canthaxanthin, and β-carotene, and 100 times greater than α-tocopherol [31]. In addition to its powerful anti-oxidant activity, AST has anti-inflammatory effects by inhibiting the NF-κB signaling pathway [32]. AST has been shown to exert an inflammatory effect by stimulating nitric oxide synthase and the NF-κB pathway, with the anti-inflammatory effect of AST (100 mg/kg) found to be comparable to that of prednisolone (10 mg/kg) in a rat model [33]. In addition, AST has been shown to be safe in humans [34]. Beyond that, AST reported reverses the metabolic syndrome, for example by decreasing insulin resistance in diabetes mellitus [35] and is a potential therapeutic agent against atherosclerotic cardiovascular disease [36].

The fact that AST inhibited glucose-induced peritoneal fibrosis was not proved directly. Several previous reports suggested that AST inhibited glucose-induced ROS and inflammation by its quenching ability [30,35],increased ROS production may directly induce TGF-β expression [37] and induce TNFα expression via JNK signal pathway [38]. Peritoneal fibrosis occurred by TGFβ via epitherial-mesenchymal transition [5] and TNF-α-induced interaction with TGF-β receptor is essential for TGF-β-dependent EMT at retinal pigment epithelial cells [39]. We previously reported peritoneal fibrosis (PF) induced by chlorhexidine (CH) was significantly suppressed in PF rats with AST supplemented diet, and the level of 8-hydroxy-2'-deoxyguanosine (8-OHdG) and cytokine such as TNFα and TGFβ were suppressed in the peritoneum[11]. According to these results, we hypothesized that AST inhibits peritoneal fibrosis (PF) through suppression of TNF/TGF expression by quenching ROS. Since ascorbic acid is an unstable and short acting ROS scavenger, it appears that there was no report about the suppressive effect in peritoneal fibrosis *in vivo*.

In the present study, AST pre-treatment did not suppress ROS in the mitochondria, but suppressed EMT production of inflammatory cytokines and growth factors as well as activation of the NF-κB pathway.

We performed comparative evaluation of the effects of ROS in EMT using AA, a common anti-oxidant. Sagun *et al*. reported that AA is transported to the mitochondria through Glut 1 and demonstrated its anti-oxidative capacity in the mitochondrial genome and membrane [40]. The anti-oxidant capacity of AA primarily affected the mitochondria. AA is a water-soluble anti-oxidant and is able to immediately deoxidate generated ROS. AA was simultaneously administered in glucose-stimulating mesothelial cells. AST is also lipid-soluble and a stable and strong ROS scavenger in both extracellular and intracellular environments. Based on these characteristics, we performed AST treatment prior to glucose stimulation. Both anti-oxidants decreased extracellular ROS and 8-OHdG expression and suppressed EMT through the inhibition of TNF-α and TGF-β expressions. Several previous reports suggested that inflammatory cytokines and growth factors induce PF. Interventions including gene transfection suppress the development of PF [3,41–43]. However, there are few reports on the role of ROS in PF development. Repetitive AST administration suppresses fibrosis in rats with chlorhexidine-induced PF. This work is important because ROS induces peritoneal sclerosis via several pathways, and scavenging ROS, stimulated by high glucose, may inhibit EMT and peritoneal sclerosis.

## Conclusions

The results of the present study demonstrate that AST pre-treatment suppressed EMT, scavenged ROS, and suppressed the production of inflammatory cytokines and growth factors as well as the activation of the NF-κB pathway. AST has anti-EMT effects through anti-oxidative and anti-inflammatory actions. Therefore, AST may represent a potential treatment for PF.

## Supporting information

S1 Fig Decision concentration of anti-oxidant solution. NO2^−^/NO3^−^ concentration in medium supernatant of each groups. The glucose concentration was 140 mM. AST-G 12 h: AST pre-treatment occurred >12 h before glucose stimulation and medium exchange. Cells were stimulated by glucose for 12 h, revealing no significant change. *: p < 0.05. **: p < 0.01. ***: p < 0.0005. ****: p < 0.0001. Error bars represent SD.
(TIF)

S2 Fig Western blotting of EMT marker and NF-κB p65 protein in the nucleus. Above: E-Cadherin, Middle: α-SMA, Bottom: NFκB p65 in nucleus. E-cadherin expression was maintained each control groups and diminished in the G3h and G12h groups. αSMA expressed only in G12h group.NFκB p65 protein in nucleus was expressed strongly in G3h and G12h groups. Each protein expression at AG and AA groups were similar to control groups.
(TIF)

S1 Table Primer design of RT-PCR. GAPDH: glyceraldehyde 3-phosphate dehydrogenase, TGFβ: transforming growth factor β, TNFα: tumor necrosis factor α,VEGF: vascular endothelial growth factor, α-SMA: alpha smooth muscle actin.
(TIF)
