# Supplementary figures and images for: Scavenging of reactive oxygen species by astaxanthin inhibits epithelial–mesenchymal transition in high glucose-stimulated mesothelial cells
Source: PLoS One. 2017 Sep 19;12(9):e0184332. doi: 10.1371/journal.pone.0184332 (PMC5604950; doi:10.1371/journal.pone.0184332)

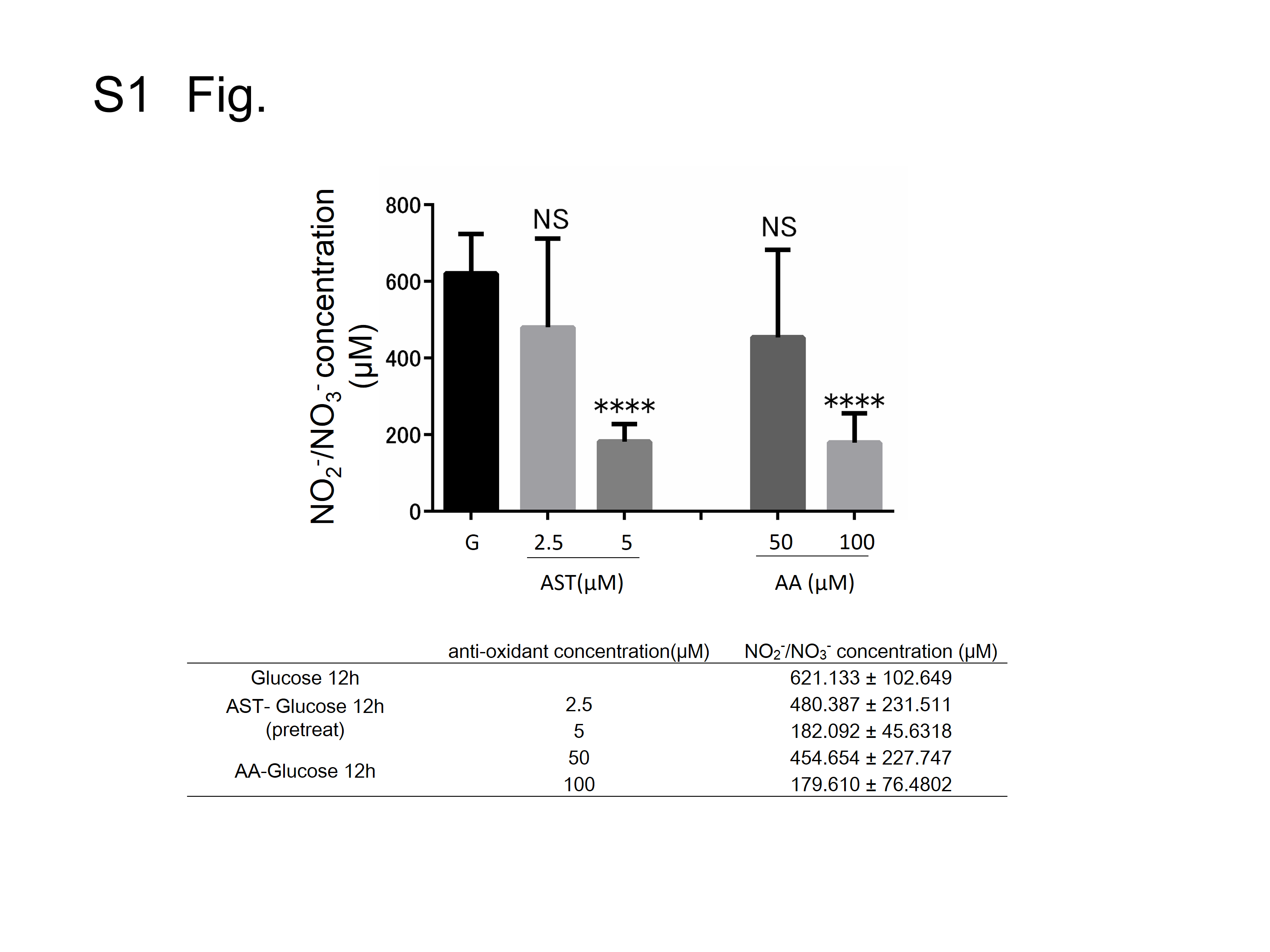

Supplement: S1 Fig — NO2−/NO3− concentration in medium supernatant of each groups. The glucose concentration was 140 mM. AST-G 12 h: AST pre-treatment occurred >12 h before glucose stimulation and medium exchange. Cells were stimulated by glucose for 12 h, revealing no significant change. *: p < 0.05. **: p < 0.01. ***: p < 0.0005. ****: p < 0.0001. Error bars represent SD. (TIF) [file pone.0184332.s001.tif]

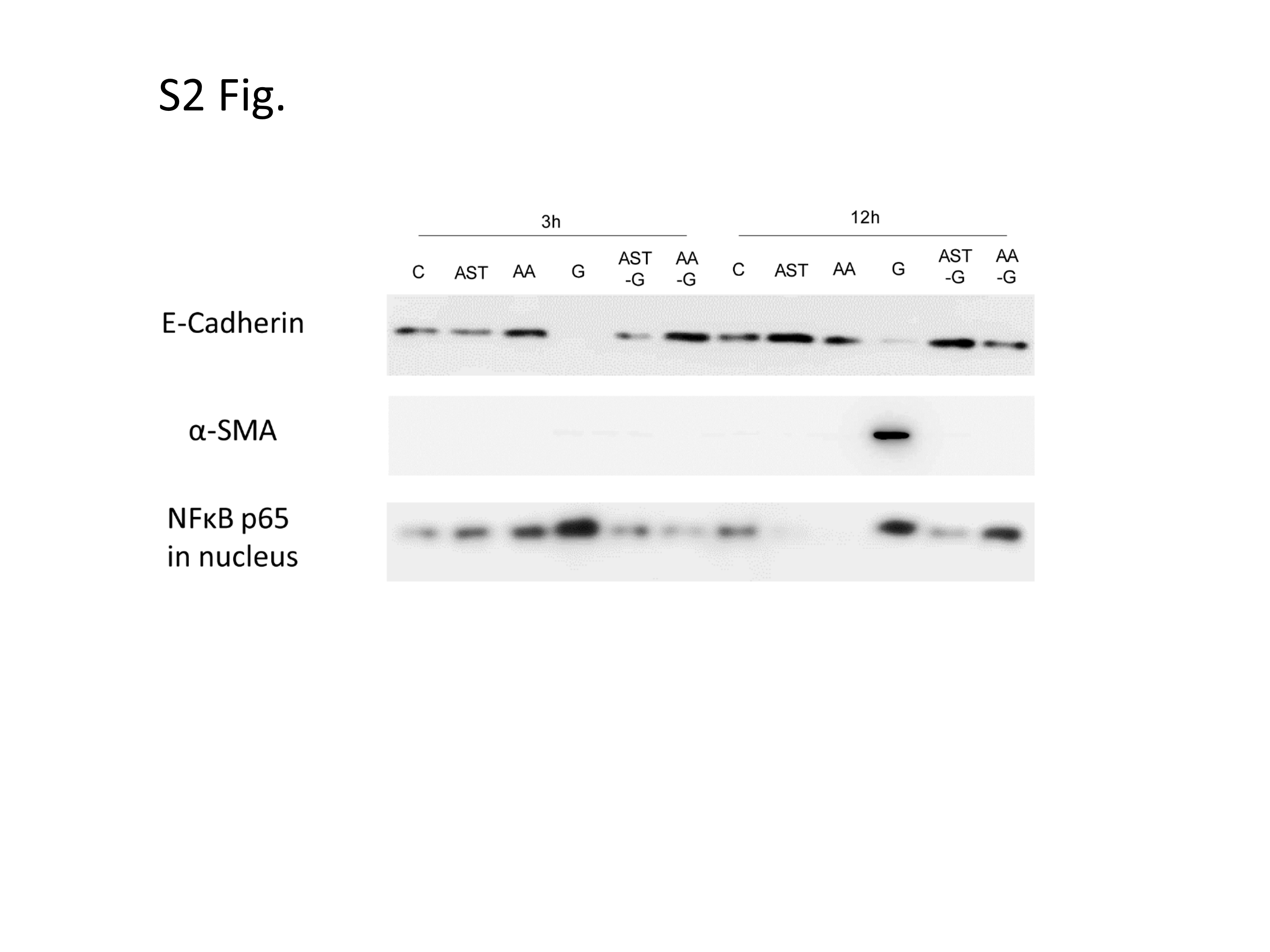

Supplement: S2 Fig — Above: E-Cadherin, Middle: α-SMA, Bottom: NFκB p65 in nucleus. E-cadherin expression was maintained each control groups and diminished in the G3h and G12h groups. αSMA expressed only in G12h group.NFκB p65 protein in nucleus was expressed strongly in G3h and G12h groups. Each protein expression at AG and AA groups were similar to control groups. (TIF) [file pone.0184332.s002.tif]

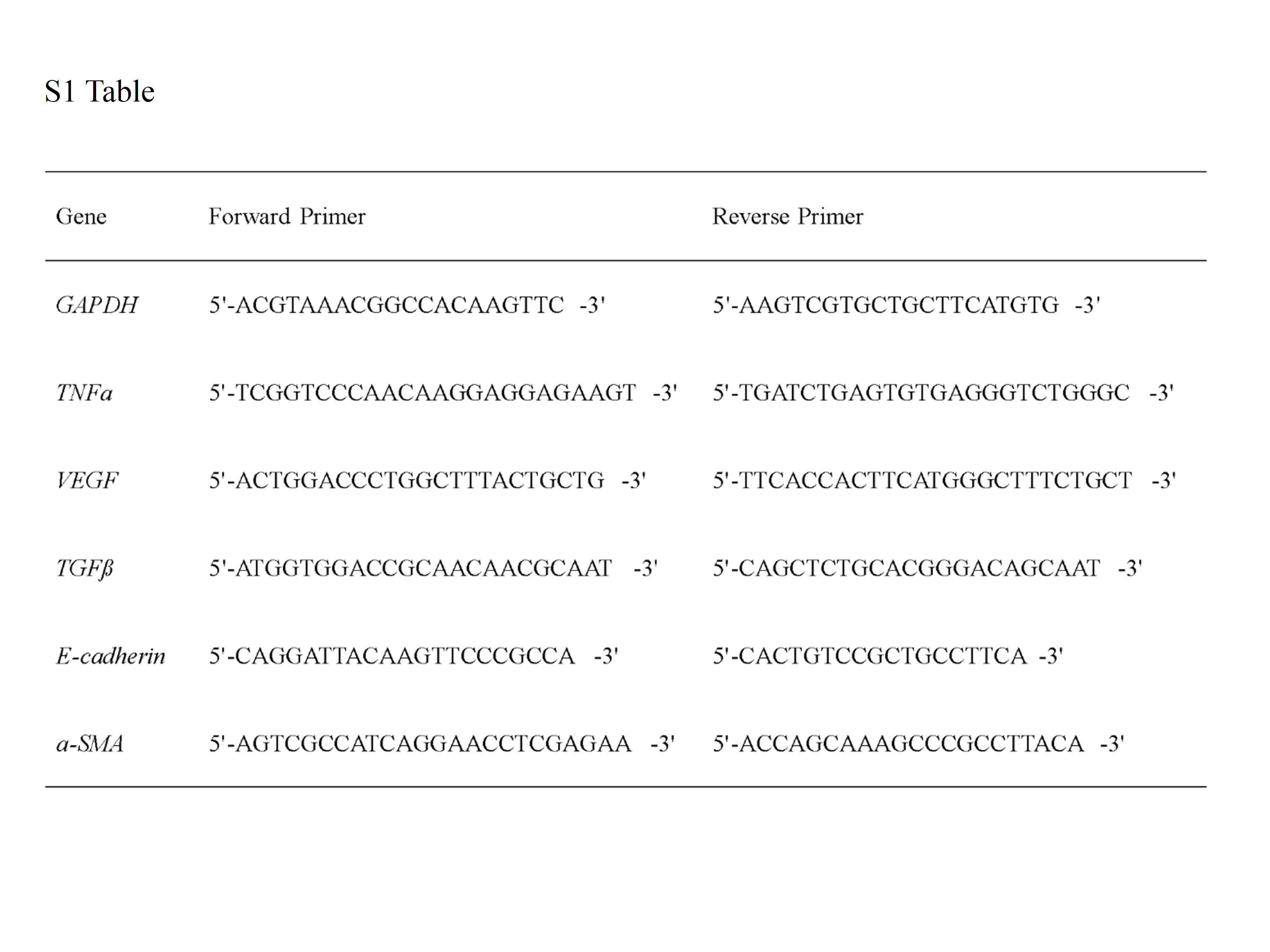

Supplement: S1 Table — GAPDH: glyceraldehyde 3-phosphate dehydrogenase, TGFβ: transforming growth factor β, TNFα: tumor necrosis factor α,VEGF: vascular endothelial growth factor, α-SMA: alpha smooth muscle actin. (TIF) [file pone.0184332.s003.tif]
